# Model to explain dental visit for children aged 0 to 5: Scoping review of birth cohorts

**DOI:** 10.1371/journal.pone.0313922

**Published:** 2025-01-08

**Authors:** Pierre-Jean BERAT, Vincent DE ANDRADE, Nolwenn REGNAULT, Annabelle TENENBAUM, Sylvie AZOGUI-LEVY

**Affiliations:** 1 Education and Health Promotion Laboratory (LEPS), (UR 3412), Sorbonne Paris-Nord University, Villetaneuse, France; 2 Pediatric Dentistry, Faculté de Santé, UFR Odontologie, Université Paris Cité, Paris, France; 3 AP-HP, Hôpital Louis Mourier, DMU ESPRIT, Colombes, France; 4 Department of Dental Public Health, Faculty of Dentistry, University Paris Cité, Paris, France; 5 AP-HP, Groupe Hospitalier Pitié Salpêtrière, Service d’Odontologie, Département de Santé Publique Orale, Paris, France; University of Minnesota School of Dentistry, UNITED STATES OF AMERICA

## Abstract

**Introduction:**

Health services accessibility is a multidimensional concept. An early-life dental visit could improve child dental health. Through birth cohorts, it is possible to identify health conditions and pathways of exposure that occur earlier in life. The aim of this study is to propose a theorical model to explain the use of dental care for children with primary teeth, based on results from birth cohorts.

**Method:**

3 databases were queried: PubMed, Embase and Dentistry & Oral Sciences Source. Eligible articles presented data on children’s dental visits, with at least one follow-up visit between birth and the child’s 6th birthday and based on birth cohorts.

**Results:**

We identified 649 articles in biomedical literature databases. After exclusions, we read 136 abstracts, and finally 36 articles in their full length. A total of 22 articles were included in the analysis, from 15 countries on 5 continents. The mains proximal factors for access to dental care for preschool children are related to caregivers’ perception of children’s oral health and its impacts on quality of life. These perceptions are influenced by the child’s oral health, the child’s and mother’s use of healthcare, and the healthcare organization. Dental fear seems to be another proximal factor. However, family social background seems to be an enabling moderator for dental visits.

**Conclusion:**

The scoping review allowed us to develop a model that explains dental visits for children aged 0–5 years as a multifactorial process influenced by caregivers’ perceptions of the child’s oral health, the family’s quality of life, and the child’s dental anxiety.

## 1. Introduction

Health services accessibility is a multidimensional concept [1–3]. Various factors influencing this ability need to be considered, such as the patient’s social and geographical environment, patient’s ability to understand the healthcare systems patient’s perceptions of his or her health needs, the time he or she is willing to invest in his or her health, the time it takes to access a healthcare facility, and perceived availability [1, 2]. Lack of use of dental care is associated with poorer child oral health [4]. Several authors have suggested different frameworks for describing and explaining the access to healthcare.

The definition of access to care has evolved. Andersen et al. identify two dimensions of access to medical care: Potential Access and Realized Access [2]. Individuals’ use of healthcare is characterized by three indicators: predisposing factors, facilitating factors and enabling factors [2]. Access to healthcare, according to Donabedian, refers to the entry into or use of the healthcare system, modulate by various barriers which limit access to healthcare [5, 6]. Furthermore, Penchansky defines "access" as a concept representing the degree of “fit” between potential patients and the healthcare providers [7]. Lastly, Levesque et al. consider the access to care as a process that begins when an individual’s healthcare needs to give rise to healthcare consequences. The transition from one phase to the next is related to five dimensions of access, and is also related to the abilities of the individuals [3].

To summarize, a review of these different models reveals that there is no single definition of access to care. However, all the authors refer to a complex process in which the need for care, and the patient’s perception of this need, is one of several factors influencing the decision to seek care. The universal models in question are useful in elucidating the issue of access to dental care, despite their original development being focused on the context of adult healthcare. There are parallels between the factors that influence access to healthcare in adulthood and those that influence access to dental care [8, 9]. Children seek dental care for a variety of reasons, including preventive care and treatment for dental pain or decay [10]. Moreover, dental caries is the most common oral disease or condition affecting the oral health of young children [11]. Unlike adults, preschool children are not autonomous in their actions. Their ability to act is dependent on the caregiver’s willingness and ability to provide care for his/her child [12].

Levesque’s framework and the Anderson behavioral model of healthcare utilization were used in a cross-sectional study to describe the determinants of access to pediatric dental care [8, 9]. The cross-sectional design of this study has limitations in determining the causal relationship between the independent variables and the outcomes [13]. So, we did not find any specific model explaining children’s healthcare use.

The use of a birth cohort study helps to limit recall bias and to better estimate the sequence of events in the child’s life [13, 14]. Birth cohorts enable us to study the successive occurrence of a social and biological processes, to study a child’s life course and his or her development within his or her environment [14, 15]. They help us to understand the natural history and causality of oral health diseases and disorders, as well as the salutogenesis actions needed to maintain and promote oral health [16, 17]. By studying children from birth, cohorts make it possible to link health events and exposures that occur early in life [17]. Oral diseases, especially the most common, early childhood caries are chronic [11]. Early intervention helps prevent caries and other oral health pathologies. An early-life dental visit could improve child dental health [18].

The aim of this study is to propose a theorical model to explain the use of dental care for children with primary teeth, based on results from birth cohorts.

## 2. Materials and methods

A scoping review was the chosen approach for suggesting a model to explain the use of dental care for children aged 0–5 years, based on results from birth cohorts. In contrast to systematic reviews and rapid reviews, which seek to provide answers to specific questions by comparing studies greater or lesser detailed and at a faster pace, scoping reviews may be designed ‘to map the key concepts rapidly underpinning a research area [19]. The objective of a scoping review is to synthesize and assess the extent of the literature on a given topic, without comparing studies [20]. The objective of a scoping review is to describe and map all the results provided by birth cohorts about children’s use of dental care.

In accordance with the recommendations for scoping reviews, our work was divided into five stages: 1) identification of the scoping review query, 2) development of the search equation to identify relevant articles, 3) selection and reading of articles, 4) graphical representation of data, 5) collation and synthesis of results [19].

We followed the PRISMA guidelines extension for scoping reviews who was developed according to published guidance by the EQUATOR (Enhancing the Quality and Transparency Of health Research) [20].

### 2.1 Search strategy

To carry out this research, we queried 3 databases (PubMed, Embase and Dentistry & Oral Sciences Source (DOSS)) using a search equation. The search equation aims to identify all the articles from birth cohorts interested in access to healthcare. In addition to keyword searches associated with birth cohorts, we included the names of known cohorts in the equation. Cohort names were retrieved from those known to the researchers and those listed by the CHICOS project for the European birth cohorts [21].

Our preliminary exploratory search has revealed that the Cochrane database Scopus and Web of Science did not yield any additional results.

The query equation was also constructed in four parts: terms related to healthcare utilization, terms associated with oral health, terms related to birth cohorts or their names if their name is known, and finally terms and filters to include only articles studying children aged 0 to 5. Additionally, a filter was employed to select English-language articles.

The initial query equation was designed for PubMed. This query equation was then adapted to the thesauri of the other databases (S1 Appendix). The search equations were designed and validated with a document resource engineer.

### 2.2 Identification of studies

This review is based on the following criteria: the population (P) is children aged 0 to 5 years; and the outcome of interest (O) is dental visits. The study design (S) is a longitudinal follow-up at birth cohorts.

Inclusion criteria consisted of the following: 1) studies must have either started the baseline data collection during pregnancy or within the first year of life or linked future oral health data to exposures during either of these 2 life stages, 2) studies described enrollment in dental services or children’s dental visits and mediators and/or moderators in the results section, 3) data were collected through at least 1 follow-up visit before 6 years of age.

Exclusion criteria comprised 1) studies published in a language other than English, 2) studies that did not collect child oral health access data, 3) studies that specifically recruited premature/low birth weight/high birth-weight children or population with other specific characteristics such as cohorts of adolescents. Cohorts generated through linked and registry data that fulfilled the above requirements for inclusion criteria were also considered in this review.

Eligible articles were those published up to September 6, 2023.

Literature reviews and congress abstracts were not included. Similarly, interventional studies embedded in cohorts were excluded.

### 2.3 Study selection

Articles identified in the electronic search were imported to bibliographic software, rayan.ai. After article identification, duplicates were removed using rayan.ai software. Titles were first screened independently by 2 reviewers. The revelant abstracts were screened with the same protocol. Full texts of relevant articles were then retrieved and examined for suitability. Any disagreements regarding the selection of studies were resolved through discussion with a third reviewer.

## 3. Results

At the end of the selection process, we included 22 articles.

**Fig 1:**
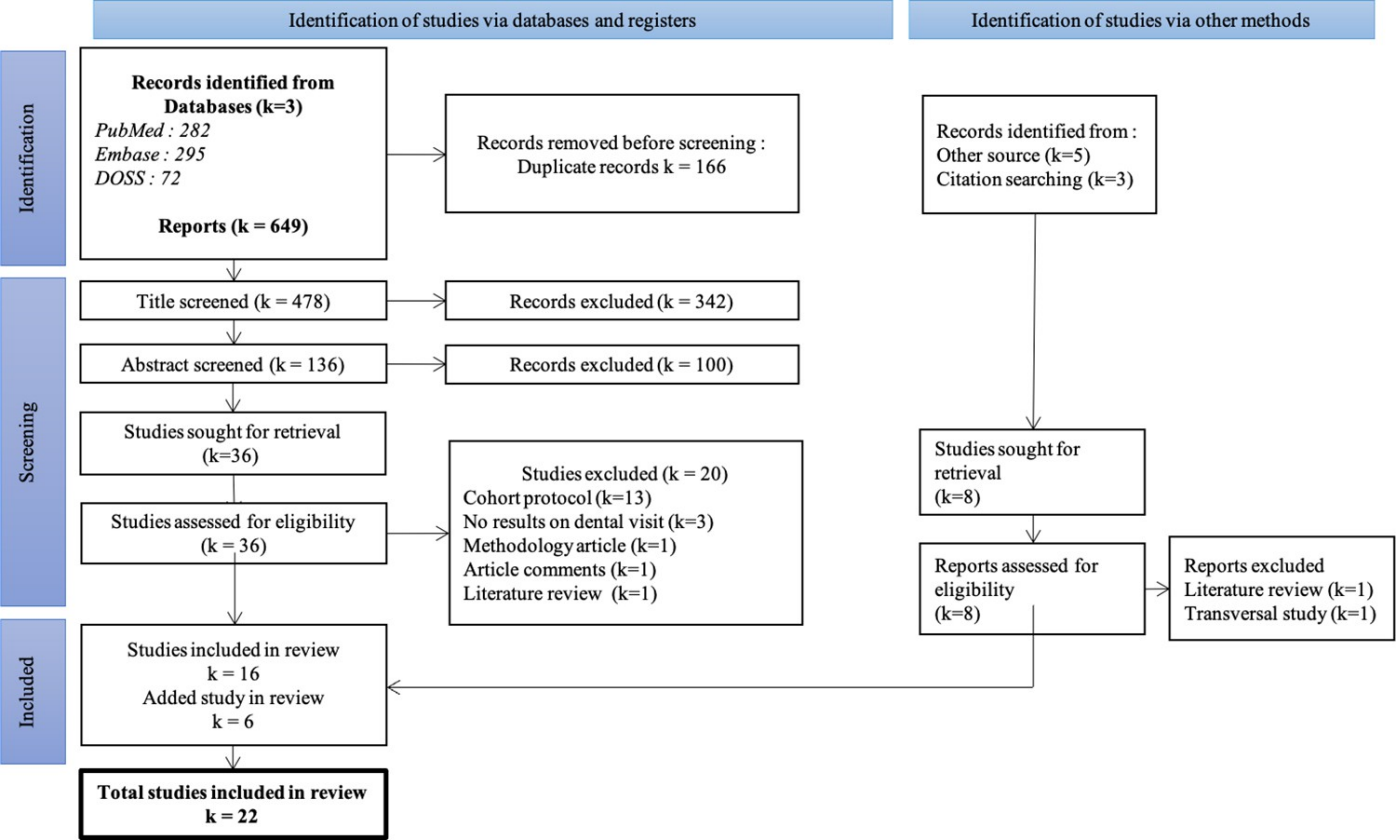
PRISMA flow chart.

The flow chart describing article selection is presented in Fig 1. Three medical databases retrieved 649 articles. After deleting duplicates, we road 478 titles. Of these, 344 were excluded, and of the remaining 136 abstracts, 100 were excluded because they did not meet the inclusion criteria. We read 36 articles in their entirety. Related research based on bibliographic references and previous knowledge led us to read a further 8 articles. After reading the articles, 22 were excluded. Of these, 13 presented different cohort protocols: 11 of them included children after their first birthday, 1 article interviewed all patients retrospectively at 6 years, and 1 article created different study groups within the cohort. A further 3 articles were excluded, as they did not present any results concerning children’s use of dental care.

Overall, 22 articles were analyzed in this review. These 22 articles came from 15 cohorts spread in all 5 continents. The studies were conducted in high-income countries with well-developed healthcare systems. The only exception is Brazil, which also has a well-developed primary care system with a high dentist population ratio [13, 18, 22–24].

They were published between 1982 and 2022. Only 5 articles were published before 2012 and study birth cohorts of children born before 2000 [10, 25–28]. The main results are presented by cohorts in Table 1.

**Table 1:** Summary of selected studies.

	Cohort Name Country (City or District) recruitment years	Children in study	Access to dental care is studied as	Main results
[25]	Christchurch Child Development Study New Zealand (Christchurch), 1977	1 084	outcome variable	The use of preschool dental services at age 4 is influenced by family social background, attendance at preschool education facilities, and utilization of routine child healthcare services.
[33]	PIF—Pacific Islands Families study New Zealand 2000	1 376	confounding factors.	School Dental Services enrollment increase with the age of the child. School Dental Services enrollment depends on the acculturation classification of their mother. Children whose mothers assimilate have better school enrollment than those whose mothers retain ethnic identity.
[38]	Gudaga Australia (New South Wales) 2005–2007	132	a factor with a potential association with dental caries	10% of parents are concerned about the oral health of aboriginal children at ages 2 and 20% of children at age 5
[39]	VicGen Australia (Victoria) 2008	466	a factor with a potential association with dental caries	Infant oral health problems (other than dental caries) reported by parents related to oral mucosa pathology, tongue-tie, oral hygiene, gingivitis. During the first month of life, the midwife or lactation consultant is more often involved in the oral examination of the infant.
[26–28]	IFS—Iowa Fluoride Study USA (Iowa) 1992–1995	198	one of the main outcome variable	The access to dental care is promoted by family income (lower or higher-than-average), the level of maternal education (high) or and the status of the child in the family (not being the first child). No association was established between the utilization of dental care services for children and the age of their mothers or fathers or the level of paternal education.
[10]	WIC—Special Supplemental Nutrition Program for Women, Infants, and Children USA (North Carolina), 1992	49 795	outcome variable	Every year, 7% of children use dental care (6% for preventative dental visit, 2% for restorative dental visit, 1% for emergency dental visit). Utilization of oral health services increased by: enrollment and participation in a public health program, Medicaid enrollment, favorable dentist-to-population ratio and being non-White Preventive or restorative dental visits increased by: access to a dental network through participation in a public health program.
[31]	COHL—The Carolina Oral Health Literacy USA (North Carolina), 2008	1 277	the main independent variable	Dental utilization is not a significant predictor of oral health literacy.
[29]	Medicaid USA (Iowa) 2000	6 322	one of the main outcome variable	Preventive well-baby visits between the ages of one and three, or the use of unique medical providers for well-baby visits, was associated with earlier first dental visits for young Medicaid-enrolled children. A greater number of preventive well baby visits between the ages of 1 and 3 or the use of unique medical providers for well-baby visits, non-white children, or children whose mothers used preventive dental care prenatally are associated with an earlier first dental visit for young Medicaid-enrolled children.
[13, 22, 23]	Pelotas Brazil (Pelotas) 2004	1 303	mediators—children’s characteristics and for one study outcome variable	A total of 51.7% of children had been diagnosed with no tooth decay. 30% of all dental visits are classified as emergency dental visit. Dental visits for children before the age of 5 increased with higher economic status, higher mothers level education and with mothers who had received guidance about prevention, attending day care. Conversely, there was a negative correlation between the frequency of children’s dental visits and the mother’s dental fear, as well as maternal tooth loss due to caries. There is an association between children dental visits and, mother’s perception of children’s oral health as good or very good, received information about prevention, child received help brushing teeth, adequate childcare, and the child is not afraid of going to the dentist. Children dental visits to the resolution of problems increased by: young mother, felt pain in the previous six months, high dfmt, perception of children’s oral health as bad or very bad. A dental visit can lead to a change in brushing habits, which can in turn reduce the tooth decay and fear of dentistry in children. Dental fear affects 17% of children by age 5. This fear can manifest in a number of ways, including a lack of dental visits for children or emergency dental visits, the child’s perception of dental pain, and the mother’s dental history. No relationship between child’s dental visit and child’s sex
[18]	Pelotas Brazil (Pelotas) 2015	2 387	outcome variable	A first dental visit before the age of one is typically for preventive reasons, as indicated by the responses of 90.9% of infants, for the resolution of issues that do not cause pain (identified by 5.4% of infants) or for the treatment of pain-related issues (identified by 3.7% of infants). A correlation was observed between maternal dental visits within the past year and maternal age, with older mothers being less likely to visit the dentist. Additionally, maternal tooth loss was inversely associated with dental visits.
[24]	Brazil (Porto Allegre) 2008	435	outcome variable	The prevalence of dental caries and dental trauma among children aged 3 years was 40% and 31%, respectively. Socio-economic inequalities in access to healthcare were partially corrected through socio-educational support from the prenatal period. Additionally, it is positively associated with higher levels of maternal education and family social class. Furthermore, it is positively associated with.
[32]	LSB21—Longitudinal Survey of Babies in the 21st Century–Japan 2001	35 260	exposure variable	Inequalities in access to dental healthcare increase with the age of the child
[36]	OMCHS—Osaka Maternal and Child Health Study Japan (Osaka) 2001–2003	315	variable	Approximately 40% of children have a regular dental check-up at age 3
[37, 40]	GUSTO–Growing Up in Singapore Towards healthy Outcomes cohorts Singapore 2009–2010	535	risk factors for ECC	Visit to the dentist (curative visit) between 9 and 24 months of children associated with the presence of tooth decay and plaque. There is no link between the mother’s dental check-up and the presence of caries lesions in the child at age 2 or 3
[30, 34, 35]	Smile for Life Belgium (Flanders) 2003–2004	722	outcome variable	A social gradient was observed for dental attendance. Child’s dental visits increases with: child’s age, level of maternal education, history of children’s dental pain, children who were not first-born, children raised by both parents, regular dental check-ups with parents, children’s compliance with oral hygiene rules.

The use of dental care is the variable that 14 studies aim to explain [10, 18, 22, 24–34]. For 9 included studies, dental care utilization was considered an exposure rather than an outcome variable [23, 34–40]. The same statement should be added in relation to the paper that investigated dental care as an explanatory variable for dental fear [13]. These papers were included as they provided valuable insights into the factors influencing the use of dental care, contributing to the construction of a model to explain the use of dental care.

In these studies, the use of dental care is explained, on the one hand, in terms of socio-demographic characteristics including psychosocial skills, and on the other in relation to health. We conclude the presentation of the results by developing a theoretical model to explain dental access for children aged 0 to 5.

### 3.1 Socio-demographic characteristics

Children’s use of dental care increases with their age (Table 2). Before the age of 1, children’s access to dental care is between 2% and 10% [18, 26, 28, 29, 38]. By age 5, between 12% and 89% of children have had at least one dental visit [10, 13, 22, 23, 25, 26, 28, 30, 35, 38]. Children who have had an early dental visit may have several consultations before the age of 5 [10, 27, 30, 32].

**Table 2:** Access to dental care by age.

	Cohort Name	Frequency of dental care visit
[25]	Christchurch Child Development Study (New Zealand)	• At least one utilization of dental care services for 83% of children at age 4
[33]	PIF (New Zealand)	• At age 2, 35.8% of children enrolled in school dental services • At age 4, 74.8% of children enrolled in school dental services • At age 6, 94.7% of children enrolled in school dental services
[38]	Gudaga (Australia)	• At the age of 2 years, 5% of Aboriginal preschool children had their first dental visit and 20% at the age of 5 years.
[39]	VicGen (Australia)	• Before 3 months, no child is examined by a dentist • At 18 months 6% examined by a private dentist and 2% by a public dentist
[26–28]	IFS (USA)	Dental visit for 89% of children in the first 5 years: • 2% before 1 year, • 32% before 3 years, • 71% before 4 years.
[10]	WIC (USA)	• 12.1% of children under age 5 use of oral health services
[29]	Medicaid (USA)	First dental examination: • 2% before 1 year, • 25% before 3 years, • 10% of children after age 3, but before age 41 months, • 35% of children, after 41 months of age. Nearly 30% of children had no first dental examination before the age of 5.
[32]	LSB21 (Japan)	• Less than 10% of children aged 2.5 received caries treatment • Between 31.5% and 41.5% of children aged 5.5 years received caries treatment, depending on their parents’ level of education.
[36]	OMCHS (Japan)	• Regular dental check-ups for 40 of the children at age 4
[30, 35]	Smile for Life (Belgium)	• Dental attendance for 38% of children before age 3 • Dental attendance for 79% of children before aged 5
[13, 22, 23]	Pelotas 2004 (Brazil)	•37% of preschool children -> at least one dental visit before age 5 (after age 2 for 67% of them)
[18]	Pelotas 2015 (Brazil)	• First dental visit before 1 year infants (average = 7 months)
[24]	(Porto Allegre) (Brazil)	• Dental visit for 26% of children by age 3

In contrast, parental age seems to play an inconsistent function in children’s use of dental care. According to some studies, the age of the parents does not seem to be associated with the child’s use of dental care, while others suggest younger maternal age correlates with fewer visits [10, 18, 22, 25, 26].

Family circumstances, such as single-parent households or frequent relocations, are linked to reduced dental care use [25, 30]. However, sibling status does not affect dental visits before age 3, although first-borns are less likely to visit a dentist before age 5 [26, 27, 30]. The child’s gender does not significantly influence dental care access [22, 30].

Moreover, preschool children who watched <1 hour of television per day were more likely to have dental care before the age of 5 [30].

The relationship between income and early dental visits is not linear; both high- and low-income families are more likely to have early dental visits [26]. Disparities in dental care utilization emerge around age 4, with higher-income families more likely to access dental care [10, 22, 24, 27, 28, 32].

Children of mothers with higher educational levels are more likely to receive dental care, a trend that has been observed more recently with paternal education levels as well [22, 24–28, 30, 32, 35]. Nevertheless, the level of maternal education appears to exert a more significant influence [32].

In addition, children of immigrants or non-assimilated parents in New Zealand have limited access to dental care within the country [25, 33]. Aboriginal preschoolers in Australia, despite healthcare programs, also exhibit fewer dental visits compared to their non-Aboriginal peers [38]. In contrast, U.S. non-white preschoolers enrolled in social programs tend to have more frequent dental visits [10, 29].

Besides, living in an area with a favorable dentist-to-population ratio seems to encourage parents to seek care for their preschool children [10].

### 3.2 Heath

Young children with more well-baby visits between 1 and 2 years or between 2 and 3 years are related to earlier first dental visits. In contrast, the number of well-baby visits before age 1 year and the timing of well-baby visits were not related [29]. Increasing the number of professionals consulted during these preventive medical visits is associated with less early use of dental care for the children [29]. Likewise mothers who had multiple prenatal visits are more likely to take their children to the dentist [22].

Besides children enrolled in early school education programs, receiving early childhood preventive medical care or participating in a social health program is more likely to visit the dentist regularly, in particular for preventive reasons, before age 4. A more efficient health program would be one that involves a dental team [10, 22, 24, 25]. However, reporting a dental visit does not mean that the children received the dental care they needed [24].

Involvement in infant health prevention and support programs seems to promote the use of dental care for prevention or restoration of teeth, and reduce the risk of emergency dental visits [10].

Children’s use of dental access is influenced by mothers’ anxiety about dental care [23]. Children’s dental visits are more frequent when parents have regular dental visits themselves, both at the time of the child’s birth and thereafter [18, 22, 23, 30]. On the other hand, mothers who have recently lost a tooth due to tooth decay are more likely not to take their child to the dentist during the first year of life [18]. Mother’s education in early childhood dental prevention promotes children’s access to dental care [22].

### 3.3 Reason for dental visit

Dental access may be associated with the presence of early childhood caries. In this case, the purpose of the dental visit is curative treatment [13, 22, 23, 25, 30, 36, 37]. Children with a history of dental pain are more likely to have had dental visits before age 5 [30]. If children have experienced pain in the last 6 months, they are more likely to seek dental treatment for curative reasons [22]. A more severe impact of tooth decay in quality of life is positively associated with having consulted a dentist [24].

In addition, children’s dental fear is associated with never seeking dental care or seeking dental care in an emergency [13]. Preventive dental care visits reduce dental fears [13].

Beliefs about the benefits of long-term outcomes and parents’ beliefs about access to care are also associated with parents seeking care for their children [35]. Perceiving the child’s oral health as poor or very poor, or perceiving a need for care, is associated with the caregiver seeking dental care. But, mother perception of children’s oral health as good or very good promotes routine visits [22].

After adjusting for confounding factors such as the mother’s age, maternal education, origin, marital status, dental knowledge and sense of self-efficacy, the child’s use of dental care does not seem to be related to the mother’s level of oral health literacy [31].

In the first three months of life, during well baby visits, none of the children had a mouth examination by a dentist or other trained oral healthcare professionals prior to the diagnosis of their dental caries later in life. This oral examination is usually performed by a midwife or breastfeeding/lactation consultant. Sometimes this examination is performed by a hospital nurse, a speech pathologist or staff at breastfeeding clinics [39]. At 3 and 5 years of age, less than 1% of children are referred to a dentist by a family physician or paediatrician [30].

Parents reported that their infant had some oral health problems (other than tooth decay) such as oral mucosa disease, tongue tie, or oral hygiene and gingivitis [39]. A history of dental trauma is another reason for dental visits before age 3 [30]. Starting at age 3, the main reason for visiting the dentist is to check up [18, 30]. Parents report their child’s first visit to the dentist as pleasant [30].

### 3.4 Dental visit explanation model

Preschoolers’ dental visits are explained by using or proposing a model in five articles. Van den Branden et al. rely on Ajzen’s Theory of Planned Behavior to explain preventive dental attendance based on parents’ beliefs and attitudes [35]. Goettems et al. present a path analysis diagram of the effects of maternal behaviors related to her oral health on the child’s use of dental care and dental caries. The child’s dental visits are therefore related to the mother’s dental visits, but also to her dental anxiety, which is linked to her perception of her oral health. In addition, the child’s use of dental visits has an effect on dental caries and brushing habits [23]. According to the theoretical model of Torriani et al., the child’s dental fear is related to his or her lack of regular dental visits. The child’s use of dental care is associated with his or her dental caries. Child’s dental caries is related to the mother’s use of dental care, her oral health, her dental fear, her level of education and the family’s income [13]. Lastly, Chi et al. propose a conceptual model based on Patrick’s model to explain the relationship between preventive well baby visits and dental visits [29, 41]. The model is organized into 5 covariates: ascribed factors (immutable individual-level variables), proximal factors (modifiable individual-level variables), immediate factors (household-level mediators between proximal and intermediate variables), intermediate factors (community-level variables), and distal factors (system-level variables) [29].

**Fig 2:**
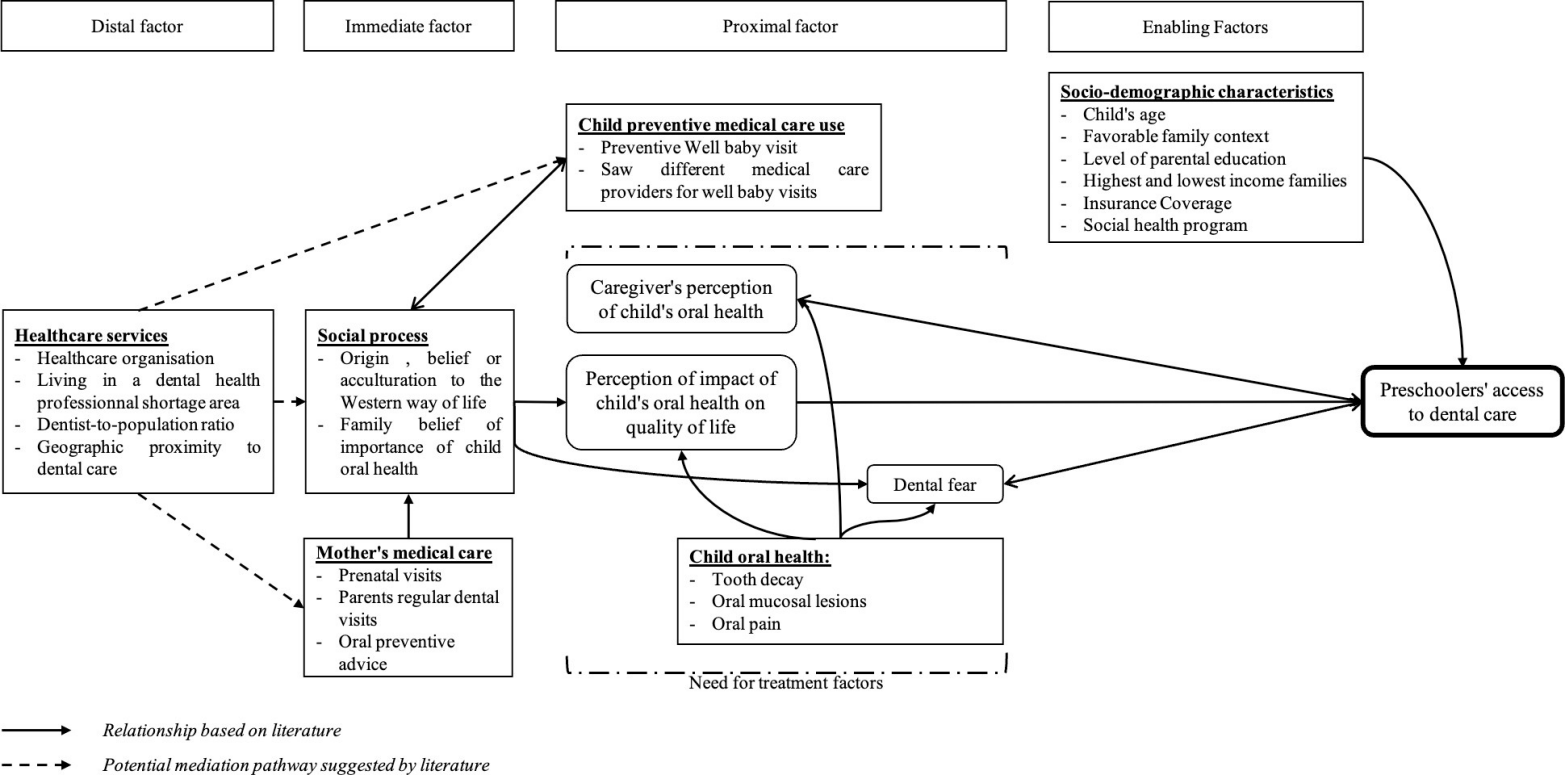
Theoretical model to explain dental access for children aged 0 to 5.

Based on various literature results and proposed models, we constructed the following model (Fig 2).

As in Levesque’s conceptual framework, the child’s access to dental care is the outcome of a process. This will depend on the supply of care and the organization of the health system (distal factors). Thus, healthcare organization, geographic proximity to the dental office, or dentist-to-population ratio influence variables related to the child’s immediate environment: parent-related factors.

The medical care for mothers, such as prenatal visits or regular dental visits by parents, is associated with family background, lifestyle, and family beliefs about the importance of children’s oral health. Therefore, the parent-related social process is related to the child’s use of preventive healthcare, such as well-child visits and the child’s consultation with various healthcare providers.

The parent-related social process (immediate factor) exerts an influence on factors related to the child’s oral health (proximal factors), in particular the caregiver’s perception of the child’s oral health and the child’s oral health-related quality of life. The child’s fear of dentistry, the caregiver’s perception of the child’s oral health, and the child’s oral health-related quality of life promote the preschooler’s access to dental care. The final two determinants are related to access to dental care and are influenced by children’s oral health. Finally, socio-demographic characteristics are factors that may or may not promote healthcare utilization. As in the Aday & Anderson framework, socio-demographic characteristics are described as enabling factors. Enabling factors describe the "means" or barriers available to individuals to use services.

## 4. Discussion

This scoping review enabled us to identify 15 cohorts in 7 countries spread over 5 continents, with varying levels of development, wealth and healthcare systems. At the end of this scoping review, we note that the majority of studies of preschool children’s use of dental care are based on cohorts of children born in the 21st century, whereas many cohorts began in the 20th century [4].

This scoping review, which focused on dental care access, identified 21 studies, whereas a previous, more general study had identified 5 studies relating to the use of dental care by preschool-age children [4]. This previous scoping review on birth cohorts showed a positive association between regular maternal dental visits and preschool children’s preventive dental visits [4, 22].

The inclusion of articles on infants and preschoolers, i.e., articles that provide data on oral health in the 0–5 age group, emphasizes early dental visits. Earlier first dental examinations are likely to help prevent early childhood caries [29].

This scoping review reveals a lack of consensus regarding the terminology used to describe access to dental care. As a result, the outcome criterion is defined in a number of ways, including as "utilization of dental care services," "dental visit," "dental attendance," "first dental visit," and "caries treatment." Nevertheless, early dental visits do not necessarily lead to care for children. Early dental visits may be limited to an oral examination and some preventive advice for parents. The requisite dental care for the children is not provided during this appointment or at any subsequent appointment [24]. This raises the question of the child’s unmet need for care.

The scoping review enabled us to develop a theoretical model to explain dental access for children aged 0–5 years. Levesque’s conceptual framework, already cited in the literature, is based on more historical models such as that of Aday and Anderson [1, 3, 9]. The developed model was based on a conceptual model proposed by Chi et al. [29].

Socio-demographic determinants are described as moderators rather than triggers for dental care use [42]. Social and structural factors complicate parents’ ability to seek dental services for their preschool children [24]. Socio-economic inequalities seem to be partly corrected by socio-educational guidance starting in the prenatal period [24]. Public social insurance coverage for patients or their parents contributes to the reduction of social inequalities in health [10, 29]. However, facilitating access to dental care for the most disadvantaged segments of the population through a dedicated, financed pathway only partially corrects the low level of dental care use by these children [38].

The caregiver’s perception of the child’s oral health, as well as the child’s quality of life in relation to his oral health, appear to be factors explaining the triggering of recourse to dental care [22, 24]. The child’s dental fear will itself modulate the use of dental care [13].

Preventive well baby visits are indicators of health-related behaviors and beliefs that may explain an indirect relationship with early dental care, which is then mediated by caregivers’ motivations, values, and preferences [29, 42]. However, the preschool child’s use of dental care is lower than his or her use of healthcare visits [22].

Although the relationship between preschoolers’ use of dental care and their living environment, or medical density has not been established, we decided to conserve these determinants in our final model [29]. The environment in which children grow up has been identified as a factor influencing their dental visits [43]. These factors are distal. The use of dental care by the child appears to be determined first by factors that are directly related to the child and his or her family [29].

One of the limits of this study is the lack of consideration given to biopsychosocial determinants. For example, the sense of coherence is supposed to be an important psychological factor. It enables people to cope with stressors and to maintain and improve their oral health, but is not studied in the cohorts [16, 44, 45]. These studies fail to identify the salutogenis factors that are beneficial to oral health and the resources that parents and children have to actively promote children’s oral health [14]. No results were found for oral health literacy, self-efficacy of caregivers, salutogenic factors such as parental locus of control, fatalistic beliefs or oral health education, although these have been validated in the literature as determinants of children’s oral health [46–48].

## 5. Conclusion

Preschoolers’ use of dental care seems a multifactorial process. Children’s use of dental care is related not only to their caregivers’ perception of their oral health, but also to the quality of life of the child and his or her family in relation to the child’s oral health. The quality of life of the child and his or her family in relation to the child’s oral health is influenced by family social process, as well as the mother’s attitude towards her oral health and her health during pregnancy. The child’s socio-demographic characteristics, although well studied, now seem to be more of a factor modulating the use of dental care by children already being followed. It would be interesting to validate this proposed explanatory model of dental care utilization with a new analysis based on a birth cohort.

## Supporting information

S1 Checklist Preferred Reporting Items for Systematic reviews and Meta-Analyses extension for Scoping Reviews (PRISMA-ScR) checklist. (DOCX)

S1 Appendix (DOCX)

S1 Annex (XLSX)
