# Supplementary material for: Model to explain dental visit for children aged 0 to 5: Scoping review of birth cohorts
Source: PLoS One. 2025 Jan 8;20(1):e0313922. doi: 10.1371/journal.pone.0313922 (PMC11709264; doi:10.1371/journal.pone.0313922)
Supplement: S1 Appendix — (DOCX) [file pone.0313922.s002.docx]

# Appendix 1

| PubMed | ("Health Care Quality, Access, and Evaluation "[Mesh] OR "Use"[TW], OR "Access*"[TW] OR "Utilization*"[TW] OR "Visit*"[TW] OR "behavio*"[TW])  AND  ("oral hygiene"[Mesh] OR "oral health"[Mesh] OR "oral hygiene"[TW] OR "oral health"[TW] OR "Dental Care for Children"[Mesh] OR "dental car*"[TW] OR "dental health services"[TW] OR "tooth diseases"[Mesh] OR "dental caries"[TW] OR "tooth disease"[TW] OR "tooth dec*"[TW] OR "home dental care"[TW] OR "dental health"[TW])  AND  (("Longitudinal Studies"[Mesh] OR "birth cohort*"[TW] OR "pediatric cohort*"[TW] OR "cohort analys*"[TW] OR "longitudinal stud*"[TW] OR "longitudinal survey*"[TW] OR "longitudinal evaluation*"[TW]) OR "pelotas birth cohort"[TW] OR "flemish environment and health study"[TW] OR "copenhagen child cohort"[TW] OR "danish national birth cohort"[TW] OR "odense child cohort"[TW] OR "northern finland birth cohort"[TW] OR "elfe"[TW] OR "babycare"[TW] OR "giniplus"[TW] OR "lisa plus"[TW] OR "rhea cohort" OR "cork baseline birth cohort study"[TW] OR "Lifeways Cross-Generation Cohort Study"[TW] OR "piccolipiu"[TW] OR "kaunas cohort"[TW] OR "ABCD-study cohort"[TW] OR "gecko drenthe"[TW] OR "generation r study"[TW] OR "koala birth cohort study"[TW] OR "mefab"[TW] OR "piama birth cohort"[TW] OR "pride study"[TW] OR "whistler birth cohort"[TW] OR "arcrisk"[TW] OR "norwegian human milk study"[TW] OR "norwegian mother and child cohort"[TW] OR "moba"[TW] OR "krakow cohort"[TW] OR "inma project"[TW] OR "bamse"[TW] OR "inuendo"[TW] OR "avon longitudinal study of parents and children alspac"[TW] OR "born in bradford"[TW] OR "ehl"[TW] OR "growing up in singapore"[TW] OR "millennium cohort"[TW] OR "dunedin multidisciplinary"[TW] OR "SMILE study" [TW] OR "Jena " [TW] OR "Hong Kong Children" [TW] OR " PROMISE-EBF study" [TW] OR "IFS"[TW] OR " Iowa Fluoride Study" [TW])  AND  ("english"[Language] OR "french"[Language]) AND (newborn[Filter] OR allinfant[Filter] OR infant[Filter] OR preschoolchild[Filter] OR "Infant"[Mesh] OR "Child, Preschool"[Mesh] OR "infant*"[TW] OR "preschool*"[TW] OR "newborn*"[TW] OR "toddler*"[TW]). |
| --- | --- |
| Embase | **#1**,"'health care facilities and services'/exp OR 'health care facilities and services' OR 'health care utilization'/exp OR 'health care utilization' OR 'health care organization'/exp OR 'health care organization'"  **#2**,"'use':ti,ab,kw OR 'access*':ti,ab,kw OR 'utilization*':ti,ab,kw OR 'visit*':ti,ab,kw OR 'behavio*':ti,ab,kw"  **#3**,"'dental car*':ti,ab,kw OR 'mouth hygiene':ti,ab,kw OR 'mouth diseas*':ti,ab,kw OR 'dental procedure':ti,ab,kw OR 'tooth disease':ti,ab,kw OR 'oral health':ti,ab,kw OR 'pediatric dentis*':ti,ab,kw OR 'dental health services':ti,ab,kw OR 'home dental care':ti,ab,kw"  **#4**,"'longitudinal study'/exp OR 'birth cohort*':ti,ab,kw OR 'pediatric cohort*':ti,ab,kw OR 'cohort analys*':ti,ab,kw OR 'longitudinal stud*':ti,ab,kw OR 'longitudinal survey*':ti,ab,kw OR 'longitudinal evaluation*':ti,ab,kw OR 'longitudinal study'/de OR 'pelotas birth cohort':ti,ab,kw OR 'flemish environment and health study':ti,ab,kw OR 'copenhagen child cohort':ti,ab,kw OR 'danish national birth cohort':ti,ab,kw OR 'odense child cohort':ti,ab,kw OR 'northern finland birth cohort':ti,ab,kw OR 'elfe':ti,ab,kw OR 'babycare':ti,ab OR 'giniplus':ti,ab,kw OR 'lisa plus':ti,ab,kw OR 'rhea cohort' OR 'cork baseline birth cohort study':ti,ab,kw OR 'lifeways cross-generation cohort study':ti,ab,kw OR 'piccolipiu':ti,ab,kw OR 'kaunas cohort':ti,ab,kw OR 'abcd-study cohort':ti,ab,kw OR 'gecko drenthe':ti,ab,kw OR 'generation r study':ti,ab,kw OR 'koala birth cohort study':ti,ab,kw OR 'mefab':ti,ab,kw OR 'piama birth cohort':ti,ab,kw OR 'pride study':ti,ab,kw OR 'whistler birth cohort':ti,ab,kw OR 'arcrisk':ti,ab,kw OR 'norwegian human milk study':ti,ab,kw OR 'norwegian mother and child cohort':ti,ab,kw OR 'moba':ti,ab,kw OR 'krakow cohort':ti,ab,kw OR 'inma project':ti,ab,kw OR 'bamse':ti,ab,kw OR 'inuendo':ti,ab,kw OR 'avon longitudinal study of parents and children alspac':ti,ab,kw OR 'born in bradford':ti,ab,kw OR 'ehl':ti,ab,kw OR 'growing up in singapore':ti,ab,kw OR 'millennium cohort':ti,ab,kw OR 'dunedin multidisciplinary':ti,ab,kw OR 'smile study':ti,ab,kw OR 'jena':ti,ab,kw OR 'hong kong children':ti,ab,kw OR 'promise-ebf study':ti,ab,kw OR 'ifs':ti,ab,kw OR 'iowa fluoride study':ti,ab,kw"  **#5**,"([infant]/lim OR [newborn]/lim OR [preschool]/lim OR 'preschool':ti,ab,kw OR 'infant':ti,ab,kw OR 'newborn':ti,ab,kw OR 'toddler':ti,ab,kw OR 'preschool child'/exp OR 'infant'/exp OR 'toddler'/exp) AND ([english]/lim OR [french]/lim)"  **#6**,"#1 OR #2"  **#7**,"#3 AND #4 AND #5 AND #6" |
| Dentistery  &  Oral Sciences Source  (DOSS) | ( (( (DE "HEALTH services accessibility") OR (DE "DENTAL care utilization") ) OR TX ( access to care or access to healthcare or access to services ) OR TX ( consult or consultation or consulting ) ) )  AND  ( (DE (Infant dental care OR Children's dental care OR pediatric dentistry OR Pediatric oral medicine OR Dental caries in children) AB (dental caries OR mouth hygiene OR mouth disease* OR dental procedure* OR dental health services OR tooth disease* OR dental care OR pediatric dentistry OR oral health OR oral hygiene) OR TI (dental caries OR mouth hygiene OR mouth disease* OR dental health services OR dental procedure* OR tooth disease* OR dental care OR pediatric dentistry OR oral health OR oral hygiene) OR KW (dental caries OR mouth hygiene OR mouth disease* OR dental procedure* OR tooth disease* OR dental care OR pediatric dentistry OR dental health services OR oral health OR oral hygiene OR Infant dental care OR Children's dental care OR pediatric dentistry OR Pediatric oral medicine OR Dental caries in children)) )  AND  ( (DE (longitudinal method) OR AB (longitudinal stud* OR longitudinal evaluation* OR longitudinal survey* OR birth cohort* OR pediatric cohort* OR cohort analys*) OR TI (longitudinal stud* OR longitudinal evaluation* OR longitudinal survey* OR birth cohort* OR pediatric cohort* OR cohort analys*) OR KW (longitudinal stud* OR longitudinal evaluation* OR longitudinal survey* OR birth cohort* OR pediatric cohort* OR cohort analys*)) OR AB ( pelotas birth cohort OR flemish environment and health study OR copenhagen child cohort OR danish national birth cohort OR odense child cohort OR northern finland birth cohort OR elfe OR babycare OR giniplus OR lisa plus OR rhea cohort OR cork baseline birth cohort study OR Lifeways Cross-Generation Cohort Study OR piccolipiu OR kaunas cohort OR ABCD-study cohort OR gecko drenthe OR generation r study OR koala birth cohort study OR mefab OR piama birth cohort OR pride study OR whistler birth cohort OR arcrisk OR norwegian human milk study OR norwegian mother and child cohort OR moba OR krakow cohort OR inma project OR bamse OR inuendo OR avon longitudinal study of parents and children alspac OR born in bradford OR ehl OR growing up in singapore OR millennium cohort OR dunedin multidisciplinary OR SMILE study OR Jena OR Hong Kong Children OR PROMISE-EBF study OR IFS OR Iowa Fluoride Study ) OR TI ( pelotas birth cohort OR flemish environment and health study OR copenhagen child cohort OR danish national birth cohort OR odense child cohort OR northern finland birth cohort OR elfe OR babycare OR giniplus OR lisa plus OR rhea cohort OR cork baseline birth cohort study OR Lifeways Cross-Generation Cohort Study OR piccolipiu OR kaunas cohort OR ABCD-study cohort OR gecko drenthe OR generation r study OR koala birth cohort study OR mefab OR piama birth cohort OR pride study OR whistler birth cohort OR arcrisk OR norwegian human milk study OR norwegian mother and child cohort OR moba OR krakow cohort OR inma project OR bamse OR inuendo OR avon longitudinal study of parents and children alspac OR born in bradford OR ehl OR growing up in singapore OR millennium cohort OR dunedin multidisciplinary OR SMILE study OR Jena OR Hong Kong Children OR PROMISE-EBF study OR IFS OR Iowa Fluoride Study ) OR KW ( pelotas birth cohort OR flemish environment and health study OR copenhagen child cohort OR danish national birth cohort OR odense child cohort OR northern finland birth cohort OR elfe OR babycare OR giniplus OR lisa plus OR rhea cohort OR cork baseline birth cohort study OR Lifeways Cross-Generation Cohort Study OR piccolipiu OR kaunas cohort OR ABCD-study cohort OR gecko drenthe OR generation r study OR koala birth cohort study OR mefab OR piama birth cohort OR pride study OR whistler birth cohort OR arcrisk OR norwegian human milk study OR norwegian mother and child cohort OR moba OR krakow cohort OR inma project OR bamse OR inuendo OR avon longitudinal study of parents and children alspac OR born in bradford OR ehl OR growing up in singapore OR millennium cohort OR dunedin multidisciplinary OR SMILE study OR Jena OR Hong Kong Children OR PROMISE-EBF study OR IFS OR Iowa Fluoride Study ) )  AND  ( (DE (Infants OR Toddlers OR Preschool children) OR AB (newborn OR infant* OR preschool OR toddler*) OR TI (newborn OR infant* OR preschool OR toddler*) OR KW (newborn OR infant* OR preschool OR toddler*)) AND LA (French OR English) ) |
